# Metformin increases PDH and suppresses HIF-1α under hypoxic conditions and induces cell death in oral squamous cell carcinoma

**DOI:** 10.18632/oncotarget.10842

**Published:** 2016-07-26

**Authors:** Talita Antunes Guimarães, Lucyana Conceição Farias, Eliane Sobrinho Santos, Carlos Alberto de Carvalho Fraga, Lissur Azevedo Orsini, Leandro de Freitas Teles, John David Feltenberger, Sabrina Ferreira de Jesus, Marcela Gonçalves de Souza, Sérgio Henrique Sousa Santos, Alfredo Maurício Batista de Paula, Ricardo Santiago Gomez, André Luiz Sena Guimarães

**Affiliations:** ^1^ Department of Dentistry, Universidade Estadual de Montes Claros, Montes Claros, Minas Gerais, Brazil; ^2^ Department of Clinical, Surgery and Oral Pathology, School of Dentistry, Universidade Federal de Minas Gerais, Belo Horizonte, Brazil; ^3^ Texas Tech University Health Science Center, Lubbock, TX, USA; ^4^ Institute of Agricultural Sciences, Food Engineering College, Universidade Federal de Minas Gerais (UFMG), Montes Claros, Minas Gerais, Brazil; ^5^ Instituto Federal de Educação, Ciência e Tecnologia do Norte de Minas Gerais (IFNMG), Araçuaí, Minas Gerais, Brazil; ^6^ Faculdades Integradas Pitágoras, Montes Claros, Minas Gerais, Brazil; ^7^ Faculdades Unidas do Norte de Minas, Montes Claros, Minas Gerais, Brazil

**Keywords:** proliferation, oral cancer, metformin, PDH, LDH-A

## Abstract

**Background:**

Metformin is a biguanide, belonging to the oral hypoglycemic agents and is a widely used in the treatment of type 2 diabetes. Evidence indicate that Metformin inhibits cell proliferation in several human cancers and inhibits the Warburg phenomenon in tumor cells.

**Results:**

Low PDH levels were observed in OSCC, and Metformin promotes an increase in PDH levels in hypoxic conditions. Metformin also reduced HIF-1α mRNA and protein levels. Metformin demonstrated antiproliferative effects, inhibited migration, increased the number of apoptotic cells and increased the transcription of caspase 3.

**Objective:**

The present study aims to explore the effects of Metformin in hypoxic conditions. Specifically, we focused on pyruvate dehydrogenase (PDH), (hypoxia-inducible factor 1α) HIF-1α levels and the oral squamous cell carcinoma (OSCC) cell phenotype. Additionally, we also investigated a theoretical consequence of Metformin treatment.

**Methods:**

PDH levels in patients with OSCC and oral dysplasia were evaluated. Metformin was administered *in vitro* to test the effect of Metformin under hypoxic conditions. The results were complemented by Bioinformatics analyses.

**Conclusions:**

In conclusion, our current findings show that Metformin reduces HIF-1α gene expression and increases PDH expression. Metformin inhibits cell proliferation and migration in the OSCC cell line model. Additionally, Metformin enhances the number of apoptotic cells and caspase 3 levels. Interestingly enough, Metformin did not increase the mutant p53 levels under hypoxic conditions.

## INTRODUCTION

Oral squamous cell carcinoma (OSCC) is a major public health problem, being the most common type of oral malignant neoplasia [1, 2]. Its treatment often produces dysfunction and distortions in speech, swallowing, mastication, dental health, and even in the ability to interact socially [3, 4]. It is one of the most frequent cancers worldwide, with approximately two-thirds of all cases occurring in developing countries [5]. Most cancer cells display a Warburg effect, a state of active glycolysis with lactate production under aerobic conditions [6–8]. Because of the Warburg effect, proliferating tumor cells consume glucose at a high rate and release lactate [9–11]. Lactate released from tissues becomes the primary precursor for hepatic gluconeogenesis [12–14]. Evidence suggests that a high serum lactate dehydrogenase (LDH) level is associated with poor survival in solid tumors [15]. Analogously to lactate, activation of (hypoxia-inducible factor 1α) HIF-1α is increased under hypoxic conditions [16–18] and promotes ATP production through increased anaerobic glycolysis [18]. This increase in HIF-1α levels under hypoxia induces OSCC invasion [19, 20]. HIF-1α also inhibits pyruvate dehydrogenase (PDH) [8]. An association between HIF-1α and OSCC was demonstrated for head and neck cancer patients' poor Eastern Cooperative Oncology Group performance status and metastasis [21–23].

Most cancer cells display a Warburg effect, a state of active glycolysis with lactate production under aerobic condition [24]. 1,1-dimethylbiguanide hydrochloride (Metformin) is a biguanide, belonging to the oral hypoglycemic agents and is widely used in the treatment of type 2 diabetes as well as polycystic ovarian syndrome, metabolic syndrome, and diabetes prevention [25]. The use of Metformin in diabetic patients is associated with a reduction in cancer incidence and mortality [26, 27]. Additionally, Metformin was associated with improved survival among diabetic patients with head and neck cancer [28]. Recent studies indicate that Metformin inhibits cell proliferation in several human cancers, including pancreatic cancer, thyroid cancer, gastric carcinoma and endometrial carcinoma [28, 29]. Furthermore, metformin and 5-FU combination therapy could exert an inhibitory effect on the Warburg phenomenon in tumor cells [30]. Metformin also effectively inhibited HIF-1α activation in cells under hypoxia [31]. Considering these facts, the present study aims to explore the effect of Metformin on PDH, HIF-1α and the OSCC cell phenotype under hypoxic conditions. Additionally, we also investigated a theoretical consequence of Metformin treatment.

## RESULTS

### Effects of Metformin on PDH and HIF-1α under hypoxia

**Figure 1:**
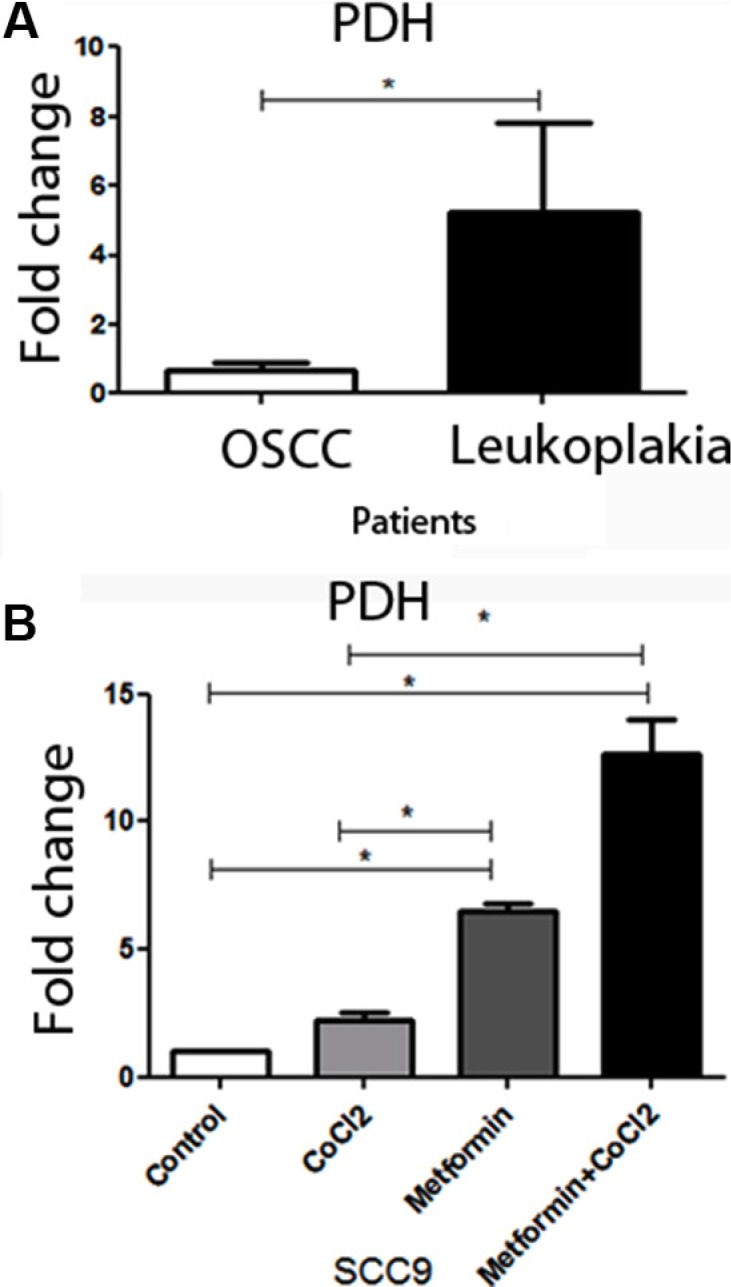
PDH levels in patients and the effect of metformin on PDH levels in SCC9 cells In (**A**), the expression of PDH in patients with carcinoma and leukoplakia. PDH mRNA levels were increased in Leukoplakia in comparison to OSCC. (**B**) The treatment of SCC9 cells increases PDH mRNA levels even under hypoxia. *Statistical significance.

**Figure 2:**
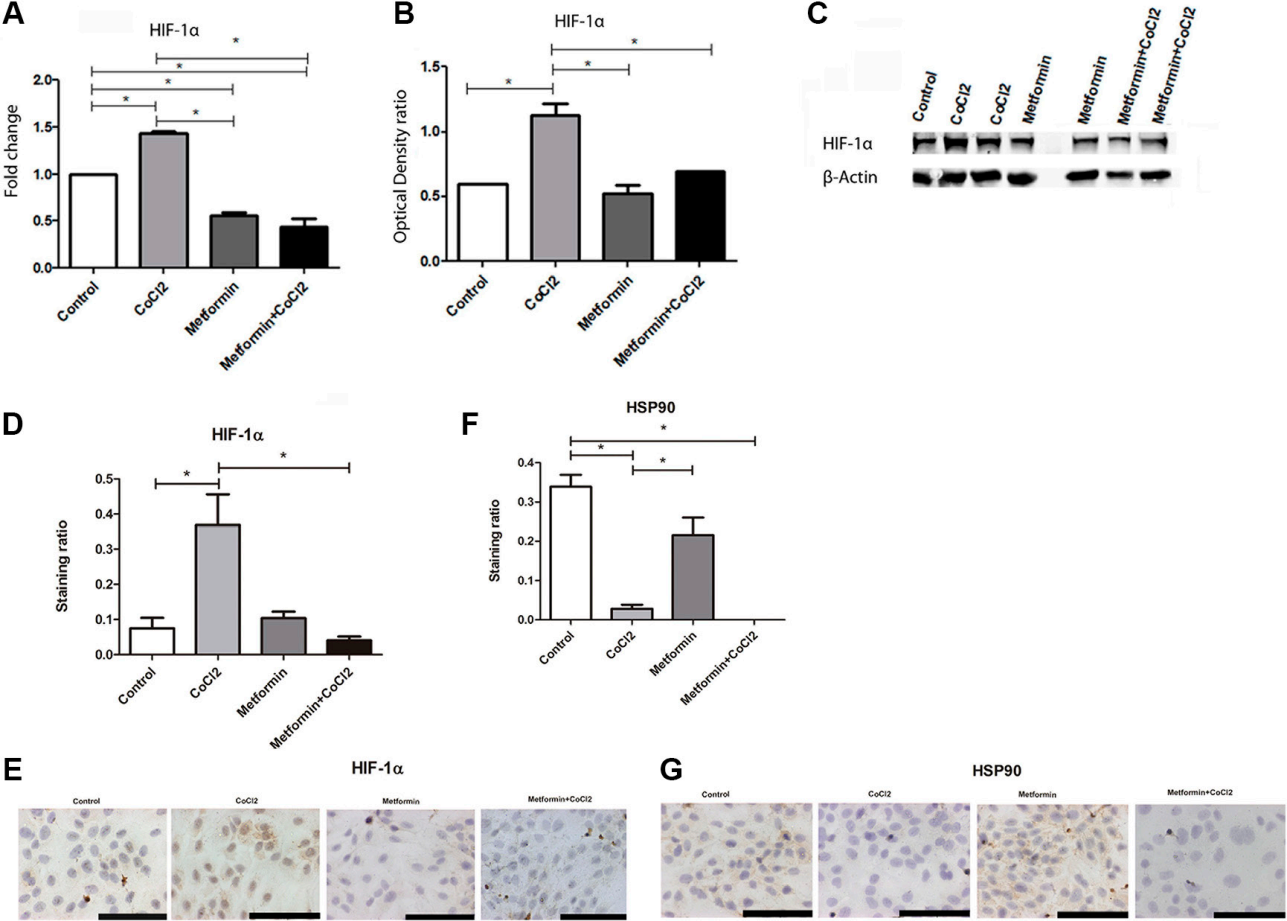
Effect of Metformin on HIF1A-1α under hypoxic conditions (**A**) Metformin reduced HIF1A-1α mRNA levels even under hypoxia. Metformin also reduced HIF1A-1α protein levels in comparison to CoCl_2_. Metformin even reduced HIF1A-1α protein levels (**B**) Quantification of optical density ratio and (**C**) Western Blot and nuclear staining (**D** and **E**). Metformin reduced HSP90 levels (**F** and **G**). *Statistical significance.

Attenuation of PDH activity could lead to HIF1α accumulation in OSCC [32]. Additionally, HIF1α increasing is related to worse prognoses of OSCC [21, 22]. Considering the role of PDH in OSCC, qRT-PCR was performed to compare PDH levels in patients with OSCC and patients with oral leukoplakia. Decreased levels of PDH mRNA levels were observed in OSCC compared to oral leukoplakia with dysplasia (Figure 1A). To test if metformin could promote an increase in PDH levels and change cells to a less aggressive cellular profile, *in vitro* assay was performed to test if Metformin could increase PDH mRNA levels in OSCC cells under hypoxic conditions. Metformin promoted an increase in PDH levels under hypoxic conditions (Figure 1B). Considering the importance of HIF-1α in anaerobic glucose metabolism and its relation to PDH [32], qRT-PCR and western blot of HIF-1α was performed to test if Metformin could change HIF-1α levels. Metformin reduced not only HIF-1α mRNA levels (Figure 2A) but also HIF-1α protein levels (Figure 2B and 2C) in SCC9 cells. Immunohistochemistry was performed to immunolocalize the HIF-1α protein. While hypoxia increased nuclear HIF-1α expression, Metformin reduced nuclear HIF-1α expression under hypoxia (Figure 2D and 2E). Evidence have demonstrated that HSP90 activity is essential for HIF-1α activation in hypoxia [33]. Metformin decreased HSP90 levels under hypoxia (Figure 2F and 2G). In the current study, HSP90 was localized only in the cell cytoplasm (Figure 2G).

### Effects of Metformin on OSCC cell phenotype under hypoxic conditions

**Figure 3:**
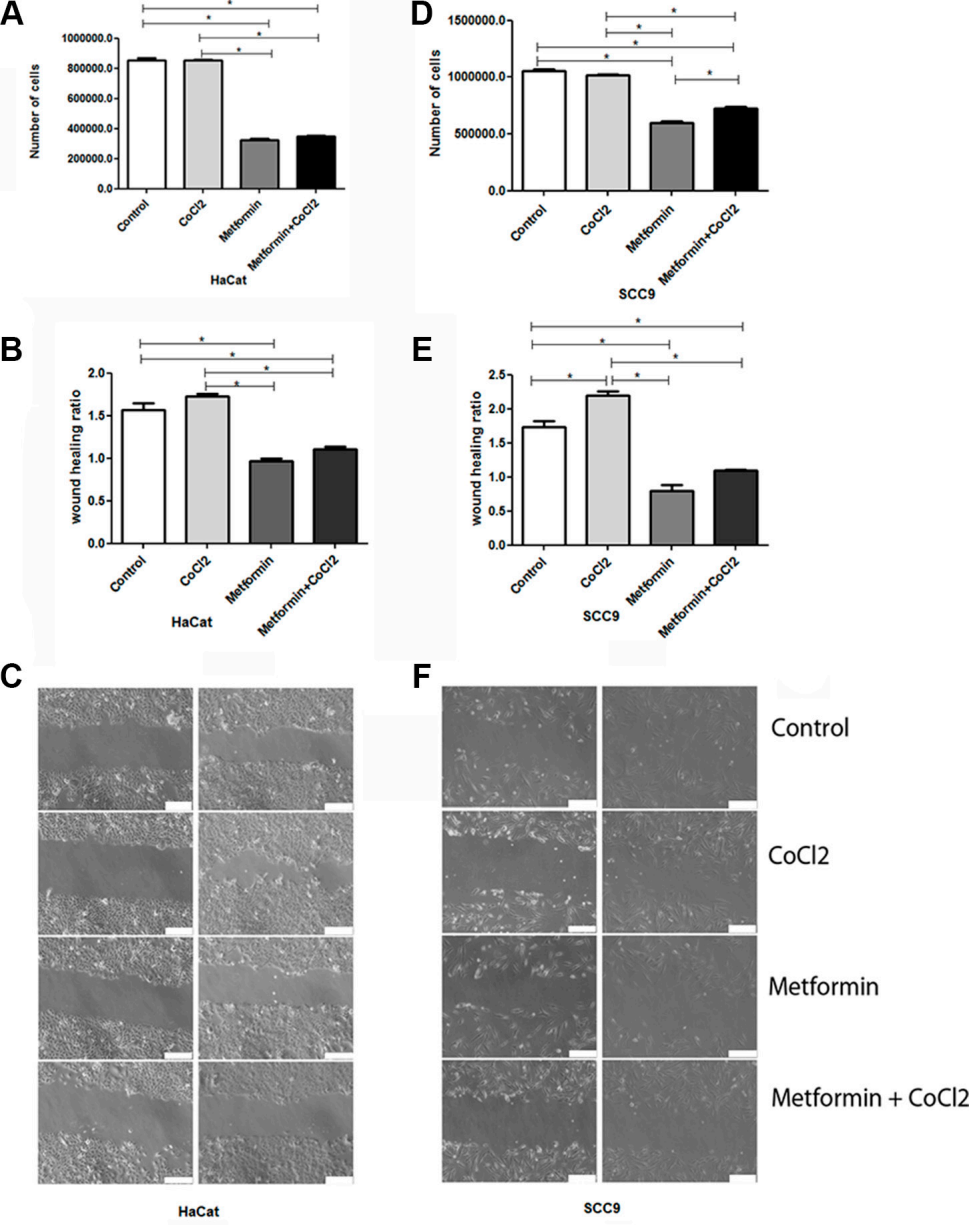
Effect of Metformin on cell death and migration under hypoxic conditions (**A** and **D**) show quantification of the effect of Metformin on the number of cells HaCat and SCC9 cells, respectively. Metformin drastically reduced the number of both cells even under hypoxia. (**B** and **E**) represent the quantification of migration of HaCat and SCC9 cells respectively. Metformin also drastically reduced cell migration ratio in both cells lineage. (**C** and **F**) illustrate wound-scratch assay of HaCat and SCC9 cells respectively. The scale of 100 μm. *Statistical significance.

**Figure 4:**
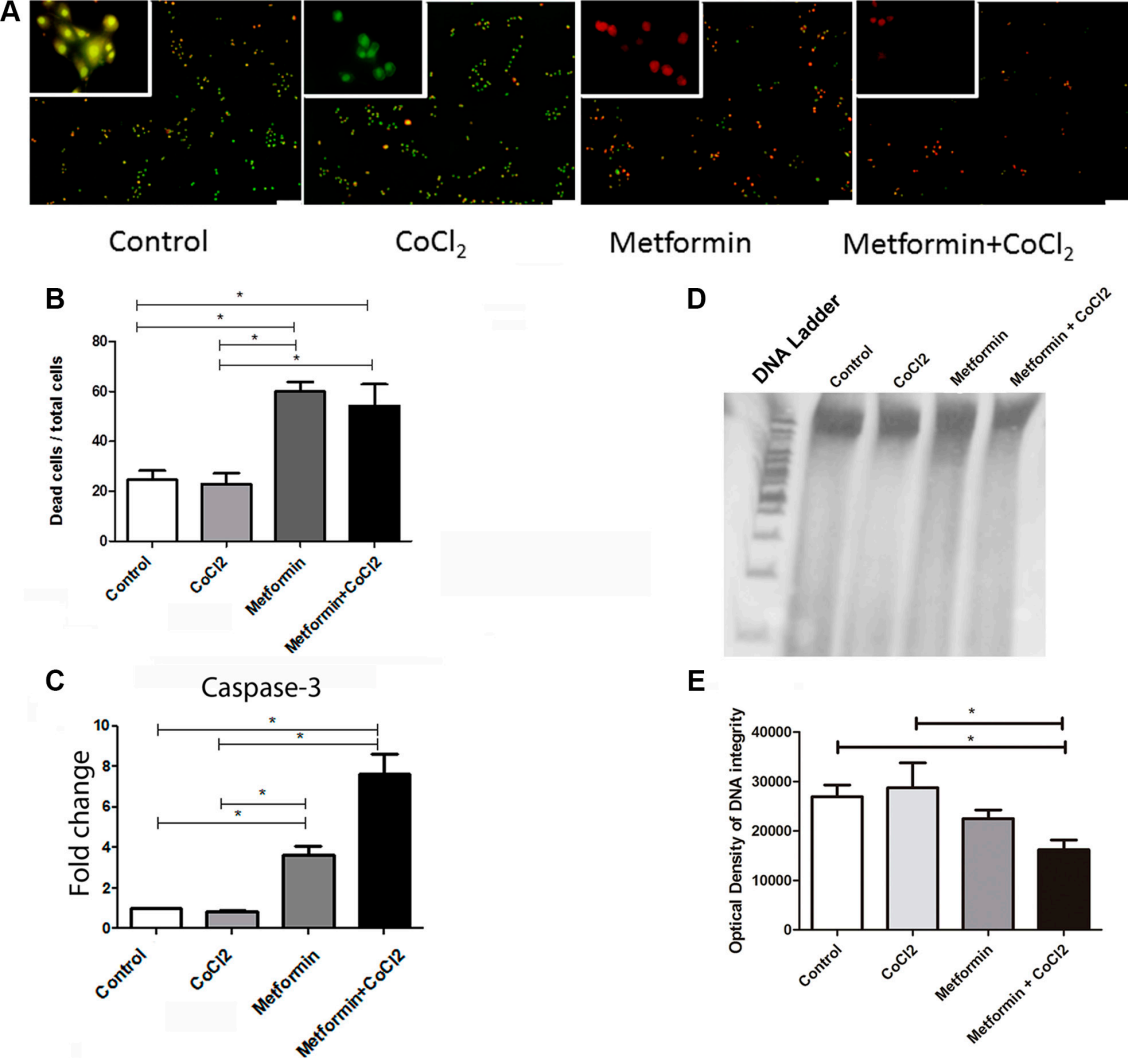
Effect of Metformin on cell death under hypoxic conditions AO/EB representative figures (**A**) and quantification (**B**) show an increase in cell death as a consequence of Metformin treatment. Metformin also increased caspase-3 mRNA levels in SCC9 cells (**C**). Metformin treatment promotes the reduction of DNA integrity in cells under hypoxia (**D** and **E**). The scale of 100 μm. *Statistical significance.

Since Metformin changes PDH and HIF-1α levels, as demonstrated before, proliferation assay, wound-scratch, AO/EB, Caspase 3 qRT-PCR and DNA fragmentation assays were performed to clarify the effect of Metformin on the OSCC cell phenotype under hypoxic conditions. Proliferation assay suggests that Metformin elicits an antiproliferative effect in immortalized keratinocytes (HaCat, Figure 3A) and OSCC (SCC9, Figure 3D). Metformin also inhibited migration significantly in Hacat (Figure 3B and 3C) and SCC9 cells (Figure 3E and 3F) according to wound-scratch assay. Additionally, Acridine Orange/Ethidium Bromide Cell death assay reveals that Metformin significantly increased the number of apoptotic cells when compared to control, even under hypoxic conditions (Figure 4A and 4B). Metformin treatment also increased the transcription of caspase 3 in SCC9 (Figure 4C). DNA fragmentation is one of the hallmarks of apoptosis. DNA fragmentation differentiates the necrotic from the apoptotic modes of cell death, and can be quantified by DNA fragmentation assay [34]. DNA of SCC9 cells under hypoxia and treated with metformin were more degraded than Control and CoCl2 groups.

### Pathways affected by Metformin treatment in the hypoxic OSCC context

**Figure 5:**
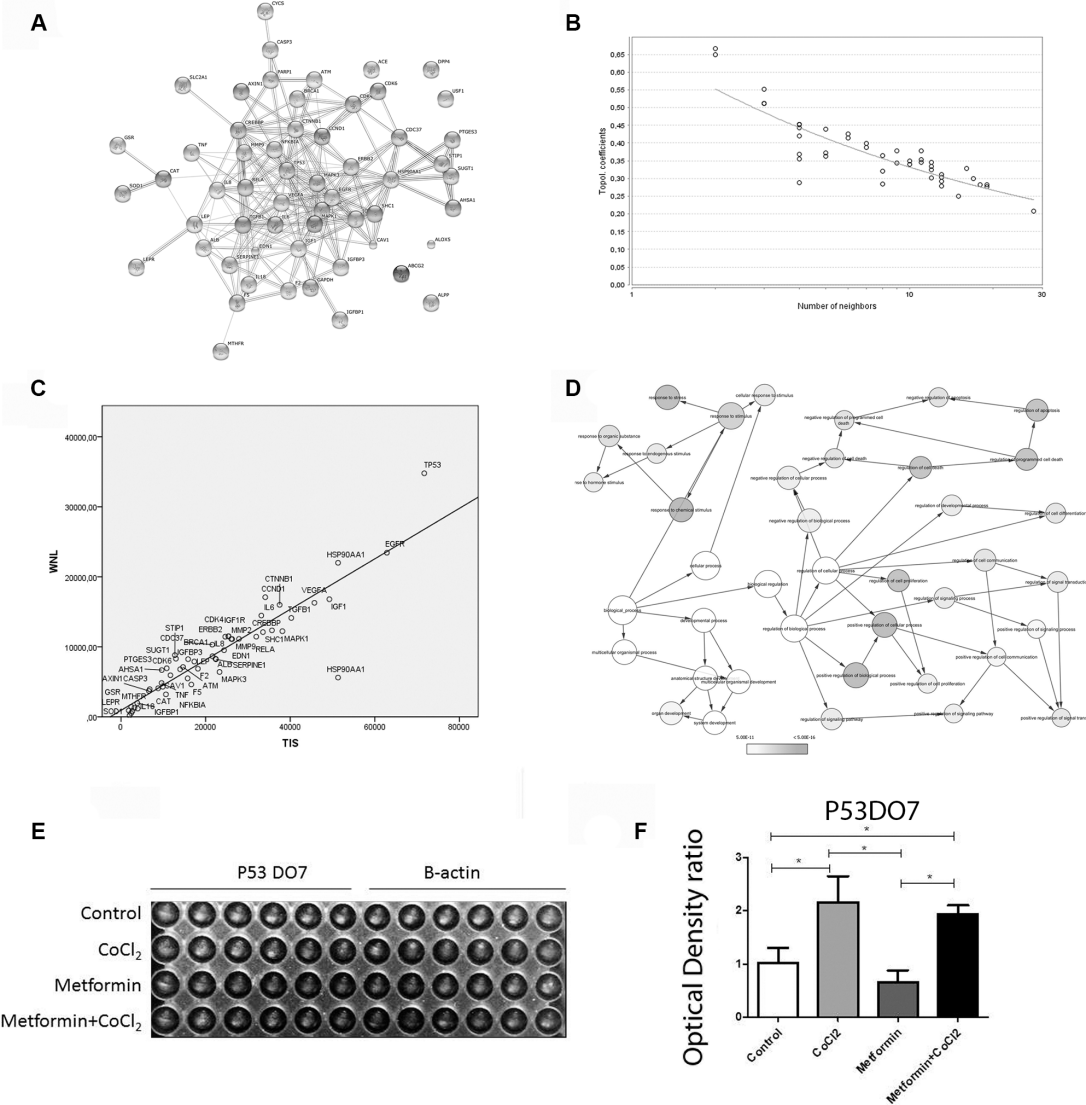
Pathways affected by Metformin treatment in the hypoxic OSCC context Bioinformatics and interaction networks analysis is demonstrated in (**A**) STRING network results, (**B**) power law behavior of the network, (**C**) Leader genes approach suggested that P53 was the leader gene and (**D**) Ontological analyses demonstrated that different mechanisms associated with death control were related to Metformin treatment. In-Cell Western Immunofluorescence Assays Chanel image (**E**) showed that metformin did not change P53 mutated protein expression (**F**) *Statistical significance.

Bioinformatics analysis was performed to evaluate the possible proteins affected by Metformin treatment in OSCC under hypoxia context. Preliminary analyses suggested 20 genes. An expansion on STRING was conducted, and all genes were included in the final network (Figure 5A). The last network exhibits a power law behavior 0.874 and R-square of 0.745 (Figure 5B). The TP53 was the protein with higher WNL and TIS (Figure 5C). The difference in WNL and TIS scores between the leader genes was confirmed by ANOVA with Tukey posthoc test (*p* < 0.001). The ontological analysis demonstrated different mechanisms associated with death control (Figure 5D). Hypoxic zones often correlate with overexpression of the mutant p53 protein [35, 36]. Therefore, we sought to investigate the effect of Metformin in mutant p53 protein levels. The DO-7 p53 clone was used to detect the mutant p53 protein. We used the In-Cell Western Immunofluorescence Assay to test if Metformin could alter the levels of the mutant protein. To simulate neoplastic hypoxic microenvironment conditions all experiments were also carried out with CoCl2. No differences between mutant p53 levels were observed with Metformin treatment (Figure 5E and 5F)

## DISCUSSION

OSCC is often treated with combination therapy using surgical resection and adjuvant radiation with or without chemotherapy [37]. The therapeutic agents are associated with severe adverse effects which cause a decrease in quality of life [38]. Therefore, identification of new useful therapeutic agents that are less toxic is crucial [39]. Oral cancer is one of the most serious health problems in many parts of the world [1, 2, 5].

Glucose is the primary source of energy and is the main fuel for cellular respiration. For glucose utilization in normal conditions, 70% of ATP is synthesized by oxidative phosphorylation and 30% by glycolysis. Evidence suggest that cancer cells might arise as a consequence of mitochondrial flaws which result in impaired aerobic glycolysis [40]. In hypoxia, glycolysis becomes enhanced to compensate the low efficiency in ATP production [51]. The high glucose consumption is known as the Warburg effect [42]. The Warburg effect is enhanced by HIF-1α inactivating pyruvate dehydrogenase (PDH). Furthermore, the inhibition of PDH diminishes the conversion of pyruvate to acetyl-CoA, allowing more pyruvate to be converted to lactate [8, 11, 41, 43]. Corroborant to these facts, in the current study, the expression of PDH in the tissues of patients diagnosed with oral carcinoma was lower when compared with oral leukoplakia, which is the most important potentially malignant lesion of the oral mucosa. Although Metformin may reduce the risk of cancer [44], the molecular mechanisms associated with the inhibitory effects on cancer development and growth are not completely understood. Interestingly enough, we found that Metformin promotes PDH expression in SCC9 cells under hypoxia.

Evidence suggest that Metformin specifically reduces HIF-1α expression and their specific target genes [24, 45]. HIF-1α is one of the markers of hypoxia, activated under hypoxic conditions, and is considered as a prognostic marker in OSCCs [21–23, 46]. HIF-1α is a heterodimeric transcription factor with α and β subunits associated with glucose uptake, metabolism, angiogenesis, erythropoiesis, cell proliferation and apoptosis [47]. HIF-1α is responsible for the expression of most of the components of glycolysis, and evidence suggests that HIF-1α has the pivotal role in the Warburg effect [43]. Interestingly enough, hypoxia increases OSCC invasion [19, 20]. In the current study, Metformin reduced HIF-1α mRNA and protein levels. Under normoxic conditions, hydroxylation leads to the degradation of HIF-1α. On the other hand, in hypoxic conditions, HIF-α subunits are stabilized and translocate to the nucleus, resulting in the activation of target genes [47]. Metformin reduced nuclear HIF-1α expression under hypoxia. HSP90 activity is essential for HIF-1α activation in hypoxia [49]. HSP90 increases bcl-2-dependent stabilization of HIF-1α protein during hypoxia [48]. Metformin decreased HSP90 levels under hypoxia.

Metformin has been associated with apoptosis promotion by increasing caspase 3 activation [49]. Activation of caspases 3, the key intracellular molecules involved in apoptosis execution [50]. Metformin is associated with a decrease in the incidence of cancers in patients with type 2 diabetes compared to patients who received other antidiabetic therapies [51]. In the current study, Metformin reduced migration and cell proliferation in SCC9 and HaCaT cell lines. Additionally, Metformin induces the expression of caspase 3 and the number of apoptotic SCC9 cells.

The leader gene approach could be used in different contexts and for different purposes [52, 53]. In the current study, we use bioinformatics to predict the central pathways affected by metformin under hypoxia. p53 was the gene associated with metformin treatment. The underlying mechanism related to the activation of p53 in response to hypoxia remains unknown [54]. HIF-1α and p53 appear to have opposing effects on glycolysis [55]. However, evidence suggests that hypoxia induces overexpression of mutant p53 detected by DO-7 clone and is associated with worse prognoses [36, 36]. In the current study, Metformin did not increase mutant p53 protein levels but did increase apoptosis.

In conclusion, our present findings show that Metformin reduces HIF-1α gene expression and increases PDH expression. Metformin inhibits cell proliferation and migration in the OSCC cell line model. Additionally, Metformin enhances the number of apoptotic cells and caspase 3 levels, but it did not increase mutant p53 levels under hypoxic conditions.

## MATERIALS AND METHODS

### Patients

Ethical approval for this study was obtained from the relevant Institutional Review Board (process number CAAE 31930314.0.0000.5146) and signed informed consent form was obtained from all patients. Five patients who presented oral leukoplakia with histological diagnoses of oral leukoplakia with epithelial dysplasia and ten patients with OSCC were enrolled in the current study to measure differences in PDH mRNA levels.

### Cell culture and hypoxia

SCC9 cells were maintained in Dulbecco's modified Eagles medium (DMEM/ F12, GIBCO, Billings, MT, USA), containing 10% fetal bovine serum (FBS, GIBCO, Billings, MT, USA), 400 ng/mL hydrocortisone, and antibiotic/antimycotic solution (Invitrogen, Carlsbad, CA, USA). HaCaT cells were cultured in DMEM (GIBCO, Billings, MT, USA), supplemented with 10% FBS at 37^°^C with 5% CO_2_ in a humidified air atmosphere. To obtain synchronized cultures of SCC9 and HaCat cells (1 × 10^5^), cells were seeded in a 12-well plate and synchronized for 24 hours by serum starvation and released with media containing 2% FBS. All treatments were performed in the absence of FBS.

To simulate hypoxic conditions, SCC9 and HaCat cells were cultured in media with the addition of 100 μM cloret cobalt (CoCl2, Sigma, St. Louis, MO, USA). The stock solutions of CoCl_2_ were filter-sterilized (0.22 μm). The resultant solutions were kept at 4°C and used within 24 hours for the assay. All culture experiments were performed in triplicate.

### Drug sensitivity assay and groups

Metformin (Galena, Campinas, SP, Brazil) was dissolved in water. Cell proliferation analysis was carried out on cells in the presence of increasing concentrations of Metformin by the Trypan blue, (Sigma, St. Louis, MO, USA) and various concentrations of Metformin (10, 20 and 50 μg/ml) were incubated for 24 hours (Supplementary Figure S1). The chosen Metformin concentration was 20 μM/ml for 24 h. Comparisons were among the four groups, which included control, Metformin 20 μM/mL, CoCl2, and CoCl2 + Metformin 20 μM/mL groups.

### RNA isolation and qRT-PCR

RNA was isolated using the Trizol reagent (Thermo Fisher Scientific, Waltham, MA, USA), according to the manufacturer. Total RNA was treated with DNase I, Amplification Grade (Invitrogen, cat number 18068015, Carlsbad, CA, USA) and then 1.5 μg of RNA was reverse transcribed with the SuperScript^®^ First-Strand Synthesis System for RT-PCR. (Invitrogen, cat number 11904018, Carlsbad, CA, USA). For qRT-PCR, 66 ng of the cDNA was added to SYBER GREEN reagent (Life Technologies, Carlsbad, CA, USA) with the caspase 3 [57], HIF-1α [20] and PDH specific primer/probe set (Life Technologies, Carlsbad, CA, USA); amplification was performed on a StepOne QRT-PCR System (Life Technologies, Carlsbad, CA, USA). All reactions were done in triplicate and Beta-Actin was used as an endogenous control for gene expression analysis. All primer sequences are displayed in the Supplementary Table S1. For experiments with patient tissues, the normal mucosa was used as a calibrator. [58]. For *in vitro* studies, untreated cells (control group) were used as a calibrator. The results were quantified as Ct values, where Ct was defined as the threshold cycle of PCR at which the amplified product is first detected and defined as relative gene expression (the ratio of target/endogenous). qRT-PCR was analyzed by the 2^-ΔΔCt. method.

### Western blot analysis

Proteins were extracted from SCC9 cell resolved on SDS-PAGE gels (10%), and then transferred onto nitrocellulose membranes and blocked with Odyssey Blocking Buffer 1× (LICOR Biosciences, Lincoln, NE, USA). The primary antibodies were anti-HIF-1α 115 kDa (1:1000, NB100-479, Novus Biologicals, Minnesota, MN, USA) and internal control anti-β-actin 45 kDa (1:1000, #4967L, Cell Signaling Technology, Danvers, MA, USA). The secondary was goat-anti-rabbit (1:15000, 926-32211 IgG IRDye^®^ 800, LICOR Biosciences, Lincoln, NE, USA). The blots were visualized and analyzed using the Odyssey Infrared Imaging System (LICOR Biosciences, Lincoln, NE, USA).

### Immunocytochemistry

SCC9 cells (1 × 10^5^) were seeded on glass coverslips and synchronized for 24 hours by serum starvation, then Metformin (Sigma) and CoCl2 (Sigma) were added to the culture medium. The following primary mouse monoclonal antibodies were used: anti-HIF-1α (1:100, H 6411, Sigma, St. Louis, MO, USA) and anti-HSP90 (1:100, sc-1055, Santa Cruz Biotechnology, Dallas, TX, USA). All monoclonal antibodies were incubated for 18 h at 4°C. Endogenous peroxidase was blocked by incubation with 0.03% H2O2 in ethanol for 30 min. The primary antibodies against HIF-1α and anti-HSP90 were detected using the Universal HRP Immunostaining Kit (KP-500, Diagnostic BioSystems, Pleasanton, CA, USA). Signals were developed with 3′3-diaminobenzidine-tetrahydrochloride for 5 min and counterstained with Mayer's hematoxylin for 30 sec. Negative controls were performed by replacing the primary antibody with PBS. Slides were photographed on Brightfield microscope FSX100 (Olympus, Center Valley, PA, USA) at 40×. The manual counts were performed in merge image by ImageJ software [61]. Immunocytochemistry analyses of all investigated antigens were carried out by determining the percentage of positively stained cells in all fields counted (33,859.00 μm^2^ for each group). For HIF-1α it was also considered the immunolocalization of the staining (nuclear or cytoplasmic). Only cytoplasmic HSP90 were found positive as it was recommended by the manufacture and demonstrated in OSCC samples [59].

### Proliferation assay

SCC9 and HaCat cells (1 × 10^5^) were seeded in a 12-well plate and synchronized for 24 hours by serum starvation, then Metformin (Sigma) and CoCl_2_ (Sigma) were added to the culture medium. After 24 hours cells were trypsinized and cell viability was assessed by trypan blue staining (Sigma, St. Louis, MO, USA). Cells were counted in a Newbauer chamber (prolab, São Paulo, SP, Brazil).

### Wound scratch assay

Cell migration was monitored in a wound scratch assay as described previously [20, 60]. Briefly, a scratch was made with a sterile pipette tip in a confluent cell layer, washed twice in PBS, and then Metformin 20 μM/ml and CoCl2 100 μmol were added in serum-free medium. Wells were photographed at the beginning of the experiment and after 24 hours (SCC9 /HaCat cells). Pictures were obtained with a camera SC30 (Olympus, Center Valley, PA, USA) in an IX81 inverted microscope (Olympus, Center Valley, PA, USA). ImageJ software was used for analysis [61]. To calculate the wound healing ratio, the initial area (in pixels) was divided by the final cell-free area (in pixels).

### Acridine orange/ethidium bromide cell death assay

The detection of apoptotic cells was performed by simultaneous staining with both acridine orange (AO, Sigma, St. Louis, MO, USA) and ethidium bromide (EB, Sigma, St. Louis, MO, USA). Cells were incubated in 10 μg/ml of AO and 20 μg/ml of EB on the dark room for 5 min. Cells were then mounted and observed under a fluorescence microscope FSX100 (Olympus, Center Valley, PA, USA). Intense EB (Ex360-370, Em420-460, filter DM400) staining indicates cell death, while extreme AO (Ex460-495, Em510-550, filter DM505) indicates live cells. The automatic count and threshold were performed in merge image by ImageJ software [61]. Evidence suggest that AO/EB allows for the discrimination of live, apoptotic and necrotic cells [62].

### DNA fragmentation assay

DNA was extracted from SCC9 cells as described before [63]. 620 ng of DNA were submitted to a 6.5% polyacrylamide gel electrophoresis stained with silver nitrate [64]. The gel quantification analyses were performed with ImageJ software [61]. The experiments were performed in triplicate.

### Bioinformatics and interaction network analysis

The leader gene approach was described previously [53, 65, 66]. Briefly, OSCC, Metformin, and Hypoxia were used as keywords together when searched in PubMed. Additionally, OSCC, Metformin, and Hypoxia were also examined individually in Gene-Bank and Genecards. All genes were taken together to build the biological network in STRING (version 9.05) [56]. Only experimental studies with a high degree of confidence (0.9–0.99) were considered. The initial gene list was then expanded using the Web-available software STRING (version 9.05) [56]. The STRING [56] software was used to score each interaction to build the interaction map among the identified genes. To evaluate differences among classes regarding weighted number of links (WNL) and total interaction score (TIS), the ANOVA and Tukey-Kramer post hoc tests were used. Statistical significance was a *P value* < 0.001. Interacting genes were classified as increased or decreased. The topological analysis was carried out with Cytoscape [67], while ontological analysis was performed with BinGO [68]. The leader genes were those comprised of the cluster with higher WNL and TIS [52, 53].

### In-cell western

In-Cell Western was performed as described before [69]. Briefly, Cells were plated (1 × 10^5^) cells/well 24 hours and incubated at 37^°^C. Before treatment, all wells were washed with DMEM before beginning the experiments. Cell treatments were dispensed with a calibrated digital multichannel pipette with a repeater function to ensure accuracy in treatment timing. After treatment (Control, CoCl_2_, Metformin, and Metformin + CoCl_2_), cells were fixed immediately by adding concentrated formalin to a final concentration of 3.7% formaldehyde and incubated at R/T for 10 minutes. After fixation, the plates were washed with PBS containing 0.1% Triton-X-100. Cells were blocked using 20 mL/well Odyssey Blocking Buffer for 1 hour at R/T. Primary antibodies were incubated O/N at 4^°^C 20 mL/well. Anti-p53 mouse (1:100, M700129, clone DO-7, Dako, Carpinteria, CA, USA) and internal control β-actin 45 kDa (1:5000, #4967L, Cell Signaling Technology, Danvers, MA, USA). After incubation, plates were washed 3 times (10 min) with 100 mL/well PBS-T at R/T on a shaker. Secondary antibodies for anti-p53 (goat-anti-mouse 1:15000, 926-32210, IgG IRDye^®^ 800, LICOR Biosciences, Lincoln, NE, USA) and for β-actin (goat-anti-rabbit 1:15000, 926-32211 IgG IRDye^®^ 800, LICOR Biosciences, Lincoln, NE, USA) were used for detection of antibody targets. Plates were incubated with 100 μL/well secondary antibody solutions for 90 min at R/T in the dark. After secondary antibody incubations, plates were washed 3 times (10 min) with PBS-T at R/T in a shaker in the dark and then filled with 50 μL/well PBS. In-Cell Western Immunofluorescence Assays were visualized and analyzed using the Odyssey Infrared Imaging System (LICOR Biosciences, Lincoln, NE, USA).

### Statistical analysis

Analyses were performed using SPSS (Version 18.0) and GraphPad Prism software (Version 5.0, GraphPad Software Inc., San Diego, CA, USA). Kolmogorov-Smirnov and the Shapiro-Wilk Tests were performed to evaluate data distribution. As samples presented as a normal distribution, one-way ANOVA, followed by Tukey post test was conducted. All data are given as means ± S.D. Statistical significance was accepted at *p* < 0.05.

## BULLET POINTS

- Metformin promotes an increase in PDH levels in oral cancer cells under hypoxia.
- Metformin reduced proliferation and HIF-1α levels in oral cancer cells under hypoxia.
- Metformin increases the number of apoptotic cells and caspase 3 levels in oral cancer cells under hypoxia.
- Metformin did not increase the mutant p53 levels under hypoxic conditions.

## SUPPLEMENTARY MATERIALS


