# Supplementary material for: Metformin increases PDH and suppresses HIF-1α under hypoxic conditions and induces cell death in oral squamous cell carcinoma
Source: Oncotarget. 2016 Jul 26;7(34):55057–68. doi: 10.18632/oncotarget.10842 (PMC5342401; doi:10.18632/oncotarget.10842)
Supplement: Supplementary file 1 [file oncotarget-07-55057-s001.pdf]

Metformin increases PDH and suppresses HIF-1α under hypoxic conditions and induces cell death in oral squamous cell carcinoma

Supplementary Materials

Supplementary Table S1: Primer used in real time PCR

Target	Sense	Primers for qRT-PCR
Beta actin	F<br>R	TGCCGACAGGATGCAGAAG<br>CTCAGGAGGAGCAATGATCTTGA
CASPASE 3	F<br>R	TCATAAAAGCACTGGAATGACATC<br>TTCTGAATGTTTCCCTGAGGTT
PDH	F<br>R	CCCCACAGACCATCTCATCAC<br>CCACCTCCTTTTCGTCCTGTAAG
HIF1α	F<br>R	TCTGCAACATGGAAGGTATTGC<br>CTGAGGTTGGTTACTGTTGGTATCA

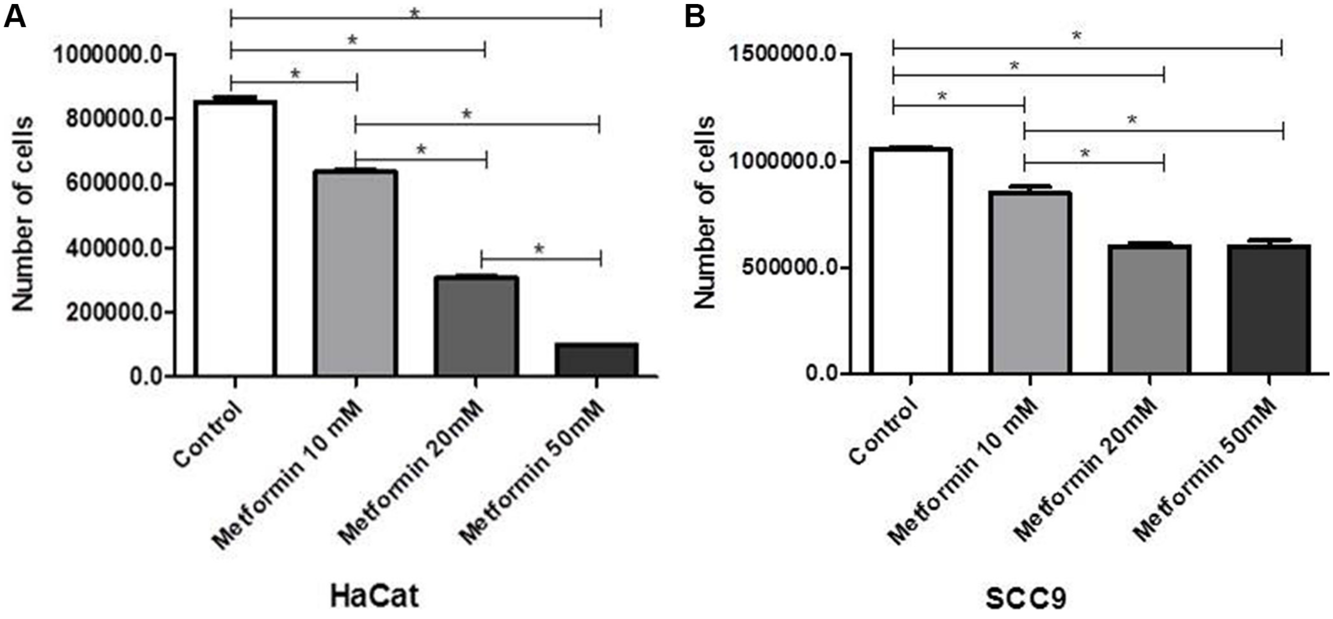

Supplementary Figure S1: Effect of various concentrations of Metformin on cell proliferation. Effect of different concentrations of Metformin on the death of HaCaT (**A**) and SCC9 (**B**) cells.
